# ItemComplex: A Python-based visualization framework for ex-post organization and integration of large language-based datasets

**DOI:** 10.1192/j.eurpsy.2025.2457

**Published:** 2025-05-26

**Authors:** Karina Janson, Karl Gottfried, Olaf Reis, Johannes Kornhuber, Anna Eichler, Michael Deuschle, Tobias Banaschewski, Frauke Nees, IMAC-Mind Consortium

**Affiliations:** 1 Department of Child and Adolescent Psychiatry and Psychotherapy, Central Institute of Mental Health, Medical Faculty Mannheim, University of Heidelberg, Mannheim, Germany; 2 Institute of Medical Psychology and Medical Sociology, University Medical Center Schleswig-Holstein, Kiel University, Kiel, Germany; 3 Department of Child and Adolescent Psychiatry, Neurology, Psychosomatics and Psychotherapy, Rostock University Medical Centre, Gehlsheimer Strasse 20, Rostock 18147, Germany; 4 Department of Psychiatry and Psychotherapy, Friedrich-Alexander-Universität Erlangen-Nürnberg (FAU), Erlangen 91054, Germany; 5 Department of Child and Adolescent Mental Health, Friedrich-Alexander-Universität Erlangen-Nürnberg (FAU), Erlangen 91054, Germany; 6 Department of Psychiatry and Psychotherapy, Medical Faculty Mannheim, Central Institute of Mental Health, Heidelberg University, Mannheim, Germany

**Keywords:** big data, constructs, content networks, data navigation, data structuring, data visualization, items

## Abstract

**Background:**

Nowadays, both researchers and clinicians alike have to deal with increasingly larger datasets, specifically also in the context of mental health data. Sophisticated tools for dataset visualization of information from various item-based instruments, such as questionnaire data or data from digital applications or clinical documentations, are still lacking, specifically for an integration at multiple levels and for use in both data organization and appropriate construction for its valid use in subsequent analyses.

**Methods:**

Here, we introduce *ItemComplex*, a Python-based framework for ex-post visualization of large datasets. The method exploits the comprehensive recognition of instrument alignments and the identification of new content networks and graphs based on item similarities and shared versus differential conceptual bases within and across data and studies.

**Results:**

The *ItemComplex* framework was evaluated using four existing large datasets from four different cohort studies and demonstrated successful data visualization across multi-item instruments within and across studies. *ItemComplex* enables researchers and clinicians to navigate through big datasets reliably, informatively, and quickly. Moreover, it facilitates the extraction of new insights into construct representations and concept identifications within the data.

**Conclusions:**

The *ItemComplex* app is an efficient tool in the field of big data management and analysis addressing the growing complexity of modern datasets to harness the potential hidden within these extensive collections of information. It is also easily adjustable for individual datasets and user preferences, both in the research and clinical field.

## Introduction

The valid and efficacious use of large (“big”) data is becoming a “hot topic” in research and clinical settings [1, 2]. Such data mostly come from large epidemiological and clinical cohort studies that are not only structured differently, but that also include the assessment of various information using multiple item-based instruments that need to be made available for valid analyses [e.g., 3–5]. This process to make valid use of the data comprises with several challenges, including difficulties in merging these different datasets. Moreover, data should be treated, i.e., discovered and reused, according to the findability, accessibility, interoperability, and reusability (FAIR) principles [1]. These principles serve as a guide for data producers and publishers to enhance the value of scholarly digital objects, including data, algorithms, tools, and workflows, for both human and computational stakeholders. As not only scientific research, but also clinical strategies and workflows increasingly depend on data-intensive methods, tools that allow for fast and valid data discovery and reuse are crucial.

The appropriate handling, management, and harmonization processes within as well as across cohorts have therefore been extensively discussed in recent publications [6–8]. While some of these aspects have been targeted through tools addressing single facets of these processes [9, 10], we may also need to add features and tools that specifically focus on visualization features to achieve a descriptive, but fine-grained overview of large datasets and that can also be used by researchers with less experience as well as clinicians. This becomes relevant due to the existence of the various instruments collected and resources implemented in and across the different studies. To overcome the lack of available organization and integration tools that address the data complexity through a fast and valid visualization framework, we developed the Python-based app framework *ItemComplex. ItemComplex* can not only be used to inform the big data harmonization process itself, but also allows the use of (harmonized) data for overview and preliminary analyses that re-inform and facilitate structuring procedures, including the harmonization, and, at the same time, enables valid and insights into the complex contents. In this framework, we integrated exploratory analysis pipelines to contribute to the process of variable selection and data merging, achieving a sufficient and valid level of data processing and resulting in the detection of understudied and hidden associations and factors to delineate novel research questions and to inform future studies. This aligns with recent investigations [e.g., 6,10], which presented a comprehensive examination of the trade-offs involved in data harmonization, delving into the intricacies researchers encounter when amalgamating or transforming data from diverse origins. While the focus of these studies and tools lies in furnishing directives solely for data harmonization efforts, our framework expands upon this discourse by demonstrating how integration of a large amount of data can be visualized for maximum overview and how this can not only facilitate harmonization efforts, but also inform initial analyses and foster new insights, particularly in the context of large complex multi-item cohort-based datasets. Through the incorporation of advanced analytical methodologies, the visualization approach contributes to the overarching objective of enhancing the accessibility and informativeness of big data across various research domains. In the following, we show the feasibility and usability of *ItemComplex* using multi-instrument datasets from different large-scale cohort studies, available in the research consortium Improving Mental Health and Reducing Addiction in Childhood and Adolescence through Mindfulness: Mechanisms, Prevention, and Treatment (IMAC-Mind); for more information, visit https://www.imac-mind.de/).

## Methods

### Identification of overlapping variables within and across studies and cohorts through data manipulations and visualization tool

**Figure 1.**
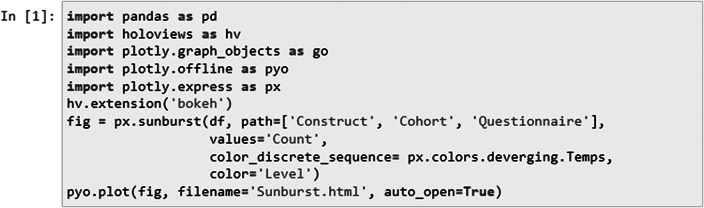
Code snippet for sunburst plot creation. The code generates a sunburst visualization displaying hierarchical data organized by levels, constructs, questionnaires, and cohorts utilizing Python libraries.

In the following, we present a user-friendly script (Figure 1), accompanied by a detailed description and guide, ensuring that even users without prior knowledge of Python programming can effectively use it. With the help of Pandas (https://pandas.pydata.org/) library, we employed data manipulation to start managing and structuring the data. With Pandas, we loaded, transformed, and organized the data into a structured format, allowing for seamless analysis. The library’s data manipulation capabilities facilitate the grouping, aggregation, and organization of data, forming the foundation for subsequent analysis.

Next, using the HoloViews (https://holoviews.org/index.htmlsimplifies) library, we then created interactive visualizations from or use case-cohort data, reducing the complexity of low-level plotting details (see the generated interactive sunburst visualization in Figure 10 [Results]). This visualization provides an intuitive representation of the hierarchical relationships among constructs/subscales across studies. With the Plotly (https://plotly.com/) library, specifically the plotly.graph_objects module, we created customizable visualizations that suit specific use case study needs. We utilized Plotly to construct the respective sunburst visualization. By customizing the color scheme and incorporating color dimensions based on specific attributes, we effectively differentiated between and identified shared constructs/subscales across studies. The chart displays hierarchical data organized by levels, constructs, questionnaires, and cohorts. The chart’s sections are colored based on different levels, and the size of each section represents a count value.

#### Data Preparation and Exploration

**Figure 2.**
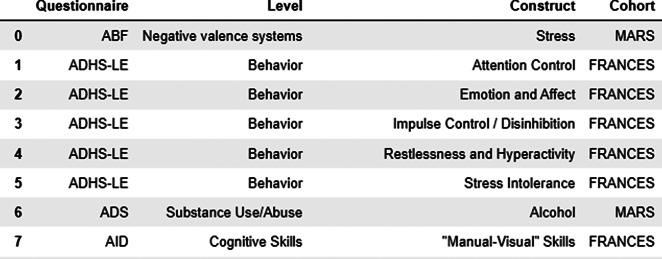
Data frame used for exploration and visualization steps.

To visually represent interconnections between constructs, instrument abbreviations, and cohorts, we used a prepared data frame (Figure 2) including the information about questionnaires used across studies.

In the second step, we utilized the px.sunburst function from the plotly.express library. The path parameter specified the hierarchical path to be represented in the sunburst chart, while the value parameters assigned the “Count” represented the sum of the cohorts (that used the questionnaire) as the source of data for each segment. We also applied a color scheme using color_discrete_sequence and distinguished segments by cohort using the color parameter.

fig = px.sunburst(df, path=[’Construct’, ’Questionnaire’, ’Cohort’], values=’Count’, color_discrete_sequence=px.colors.diverging.Tealrose, color=’Cohort’)

#### Generating and saving the visualization

After constructing the sunburst chart, we utilized the pyo.plot function from plotly.offline to generate an interactive HTML visualization that can be accessed outside of the code environment.

pyo.plot(fig, filename=’Sunburst_Plot.html’)

The filename parameter specifies the name of the generated HTML file.

### Exploring item-level relations within and across studies and cohort data through semantic similarity analyses and represented in network and graph-based visualizations

With Natural Language Toolkit (NLTK) (https://www.nltk.org/), we integrated a powerful library designed to work with human language data. We applied tools, resources, and functionalities for natural language processing (NLP), such as tokenization, stemming, part-of-speech tagging, syntactic parsing. With NetworkX (https://networkx.org/), we created, manipulated, and analyzed complex networks or graphs through a rich set of tools for working with nodes, edges, and attributes in graphs. This enabled the graphical representation of relationships and structures within our diverse items. With the TfidfVectorizer module from the *sklearn.feature_extraction.text* library in scikit-learn (https://scikit-learn.org/stable/), we converted our psychometric text documents into a numerical format suitable for machine learning algorithms. Based on the *cosine_similarity* module from the *sklearn.metrics.pairwise* library in scikit-learn, we then calculated the cosine similarity between pairs of our item-related data points to measure the similarity between items based on the cosine of the angle between their respective vector representations in a high-dimensional space (often generated using techniques like term frequency-inverse document frequency [TF-IDF]). The resulting similarity matrix provides insights into the textual relationships among different questionnaires. Cosine similarity ranges from −1 to 1, where a higher value indicates greater similarity between the vectors.

#### Data Preparation

**Figure 3.**
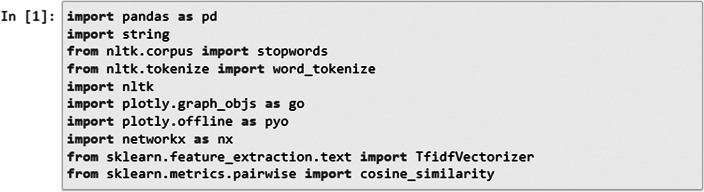
Importing libraries required for item similarity analysis.

To explore similarities on the item level, we imported the following necessary modules (Figure 3): string for punctuation manipulation, stopwords from the NLTK library for removing common English stopwords, and word_tokenize for tokenizing the text into words. First, we defined a function called preprocess_text which preprocessed a given text by performing several text-processing steps.

**Figure 4.**
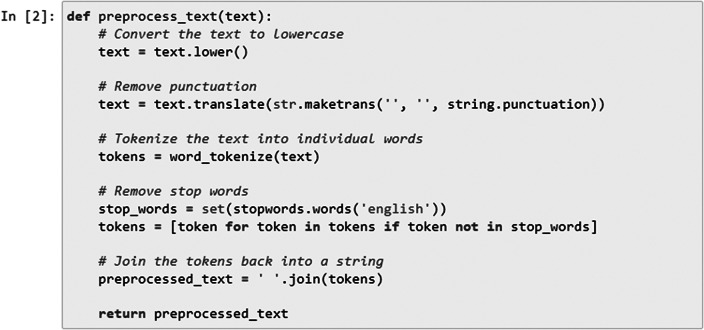
Data preprocessing for item similarity analysis.

Next, the defined preprocess_text function (Figure 4) took a single argument text, which represented the input text that had to be preprocessed. The function converted the entire text to lowercase using the .lower() method. Next, we removed punctuation from the text using the string.punctuation constant, which included all American Standard Code for Information Interchange (ASCII) punctuation characters. This was achieved by calling text.translate() with a translation table created by str.maketrans(“, “, string.punctuation).

The text was then tokenized into individual words using the word_tokenize function from NLTK. This function splits the text into a list of words. We retrieved a set of English stopwords using stopwords.words(’english’) from NLTK. This enabled then filtering out of these stopwords by creating a new list called tokens that contains only those tokens (words) that were not present in the stop_words set. Finally, the preprocessed tokens were joined back into a single string using ’ ’.join(tokens). The resulting preprocessed text was returned from the function.

## Data Processing & Cosine similarity matrix creation

**Figure 5.**
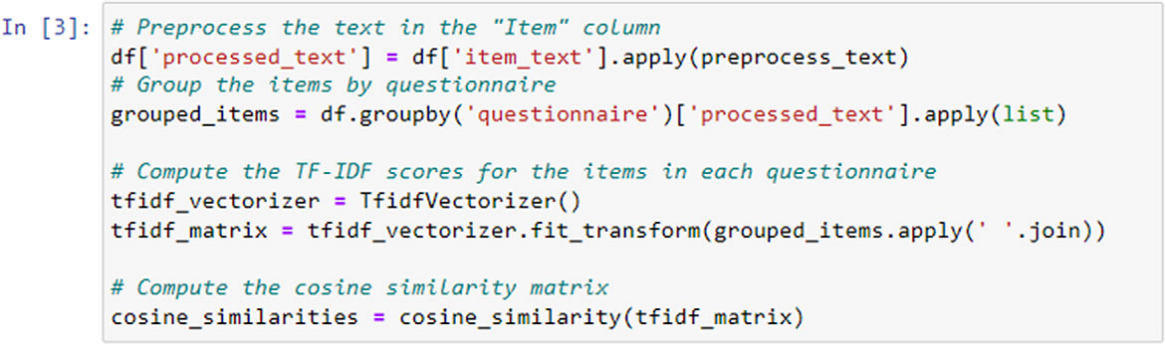
TF-IDF scores calculation.

In the following step, we applied the preprocess_text function that was previously defined to the “item_text” column of a DataFrame named df. This resulted in the creation of a new column called “processed_text” in the DataFrame (Figure 5), containing the preprocessed version of the text in the “item_text” column.

We used the TfidfVectorizer class from the scikit-learn library to convert the preprocessed text data into a TF-IDF matrix. Each row in the matrix corresponded to a questionnaire, and each column corresponded to a unique term in the entire dataset. The matrix represented the TF-IDF scores of each term within each questionnaire. We applied the fit_transform method to the preprocessed text data, where grouped_items.apply(’ ’.join) combined the preprocessed text items of each questionnaire into a single string.

The cosine_similarity function from scikit-learn computed the cosine similarity between rows (questionnaires) in the TF-IDF matrix. This results in a similarity matrix where each element (i, j) represents the cosine similarity between questionnaire i and questionnaire j.

**Figure 6.**
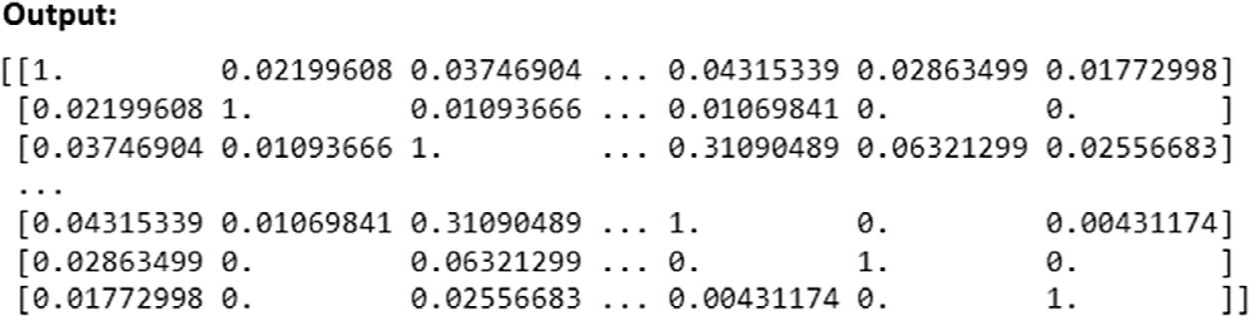
Generated cosine similarity matrix.

Finally, the cosine similarity matrix (Figure 6) was printed, showing the computed cosine similarity values between different questionnaires based on their TF-IDF representations.

### Creating the network plot to visualize the similarity between items

**Figure 7.**
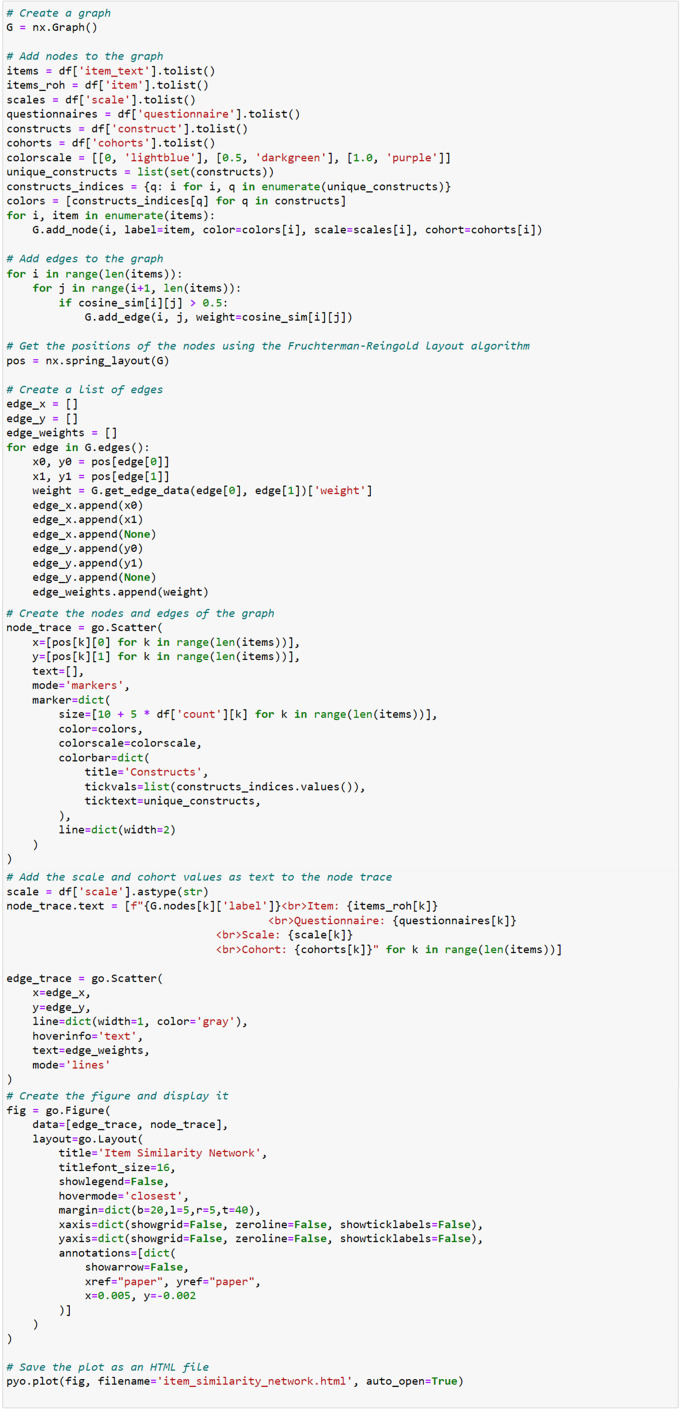
Network graph generation.

The following part of the Python script visualizes item-level similarities between survey items using the NetworkX and Plotly libraries (Figure 7). The script begins by creating an empty graph G using the nx.Graph() function. This graph will hold nodes representing survey items and edges representing relationships between them based on their semantic similarity. A list of survey items (items), along with associated data such as their scales, questionnaires, constructs, and cohorts, is retrieved from the DataFrame. Each survey item is added as a node in the graph using the G.add_node(i, label=item) command, where i represents the node index. Additional attributes like color, scale, and cohort are also assigned to each node. For each pair of survey items, the cosine similarity is checked using cosine_sim[i][j]. If the similarity exceeds 0.5, an edge is created between the corresponding nodes with a weight representing the similarity using the G.add_edge(i, j, weight=cosine_sim[i][j]) function. The spring layout algorithm (nx.spring_layout(G)) is applied to determine the positions of the nodes in the graph, ensuring that similar items (with higher edge weights) are positioned closer together.

The script then constructs a list of edge coordinates (edge_x and edge_y) by iterating over the edges of the graph, extracting the positions of each node, and storing them to be used in visualization. The nodes are visualized using go.Scatter(), with attributes such as size, color, and label text. Node sizes are determined by the count of the survey items (size=[10 + 3 * df[’count’][k] for k in range(len(items))]), while the color is assigned based on the construct of the item. The color scale is applied to visually distinguish nodes based on their construct. Similarly, the edges are drawn using another go.Scatter() function, with the edge width (line=dict(width=2)) representing the strength of the relationship between items (based on the cosine similarity score).

Finally, a figure is created using go.Figure(), which includes both the edge and node traces. The layout of the plot is configured to hide gridlines and axes, and the graph is presented as an interactive plot titled “Item Similarity Network.” The graph is saved as an HTML file using the pyo.plot() function, allowing the user to open and interact with the graph in a web browser.

*ItemComplex feasibility and usability for data from different cohort studies.* As a use case to verify the real-world applicability of ItemComplex for diverse, large-scale datasets, we employed data from four longitudinal cohort studies within the IMAC-Mind Consortium (for the interface see Figure 2). These studies span different populations, measures, and follow-up periods, allowing us to thoroughly test ItemComplex’s capacity to organize, visualize, and integrate questionnaire-based data across heterogeneous sources. Specifically, we utilized (1) severe role impairment (ROLS) [11], a cohort study that has been examining the development of children and adolescents over several decades. The study was initiated in 1991 and focused on the developmental trajectories of children from birth into adulthood. The study includes comprehensive assessments of cognitive; emotional; and social development; as well as data on environmental factors, family dynamics, and health outcomes (for more information, visit https://kjpp.med.uni-rostock.de/forschung/rostocker-laengsschnitt-studie-1). (2) MAnnheimer RisikokinderStudie (MARS) (*Mannheim Study of Children at Risk*, [12]), a longitudinal study that began in 1986 and followed a cohort of children born at high risk for developmental and mental health issues due to various prenatal and postnatal factors. The study investigated the interplay between biological, psychological, and environmental influences on the development of psychopathology and cognitive outcomes. (3) FRANCES [13], a cohort of pregnant mothers and their children from prenatal stages through adolescence examines how factors such as parental mental health, socioeconomic status, and stressful life events influence the mental health trajectories of children. (4) *Pre-, peri- and postnatal stress: epigenetic impact on Depression* (POSEIDON), a multidisciplinary research project that investigated the long-term effects of stress experienced during the pre-, peri-, and postnatal periods on the epigenetic regulation of genes associated with depression and combined data from clinical assessments, biological samples (such as DNA and RNA), and environmental measures (for more information see [14], visit https://www.neuron-eranet.eu/projects/POSEIDON/).

Recent visualization research in mental health and clinical domains has demonstrated the importance of user-centered evaluations when introducing novel tools [15, 16]. Both works integrated domain experts (e.g., clinicians, psychiatrists, researchers) into the evaluation loop, using real-world tasks and feedback sessions to validate their visual analytics systems’ usability and relevance. Following this model, we integrated ItemComplex into the IMAC-Mind Consortium’s ongoing data harmonization efforts rather than conducting a new, separate user study. Specifically, we used data from the MARS, FRANCES, POSEIDON, and ROLS cohorts as a real-world test case to verify the framework’s usability. This user involvement took place under the IMAC-Mind Consortium’s TP1 work package, wherein investigators routinely needed to navigate, harmonize, and analyze the existing cohort data. ItemComplex was introduced as the visualization and navigation tool of choice for these tasks. During consortium meetings and workshops, the integration of ItemComplex was discussed and further refined, for instance, clarifying data-upload procedures, optimizing default layouts for sunburst and TreeMap visualizations, or further refining item similarity thresholds.

## Results

**Figure 8.**
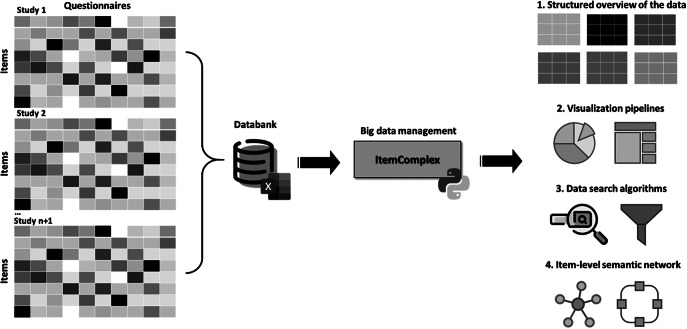
Workflow of the ItemComplex framework, a Python-based visualization methodology for the organization, structuring, and analysis of large and diverse psychometric datasets. *Note:* On the left, various large-scale cohort datasets are shown being collected and organized, emphasizing their diverse structures. These datasets are then processed through ItemComplex, which applies a suite of Python algorithms for data structuring according to content topics, along with visualization tools, including sunburst and TreeMap plots. These tools help to visually represent the hierarchical structure, overlap, and assignment of constructs/subscales within and across different studies, allowing for navigation through various levels of data granularity. Structured overview (top right): This part of the figure illustrates the structured overview process applied to large psychometric datasets. The ItemComplex framework allows for the organization and structuring of data on multiple levels, such as the questionnaire level or construct level. At the questionnaire level, the framework can identify identical or similar questionnaires across different studies, aiding in the harmonization process. At the construct level, it allows to see where the most information has been assessed, highlighting the overlaps and gaps in the data across studies. This hierarchical structuring provides a comprehensive understanding of the data landscape, making it easier to compare and integrate data from diverse sources. Visualization pipelines and data search algorithms (middle right): highlights the use of interactive data exploration pipelines within the ItemComplex framework. These pipelines are equipped with intuitive, interactive charts like sunburst and TreeMap plots, which allow users to explore the data in a user-friendly manner. By interacting with these visual tools, users can easily navigate through the data. Item-level semantic network (bottom right): The final part of the figure focuses on the item-level semantic network analysis performed by the ItemComplex framework. Using NLP techniques and cosine similarity measurements, the framework identifies semantic similarities between individual survey items across different studies. The results are visualized in network graphs that represent the relationships between items.

*The ItemComplex principle.* The Python-based *ItemComplex* app framework is sketched (Figure 8) and is developed for structuring, network, and graph analyses of multi-item-based psychometric data. Here, psychometric data refers to the quantitative information obtained from (neuro)psychological and clinical assessments and instruments applied to measure individuals’ cognitive, emotional, and behavioral attributes and responses. These data are collected through standardized behavioral tests, questionnaires, surveys, and interviews and are used to evaluate various psychological constructs such as information processing, personality traits, mental and physical health status, and other psychological facets.

The core of the *ItemComplex* app entails creating and implementing a) data search algorithms and related visualization pipelines to get a valid and quick overview of the data, and b) item-level semantic network and graph analyses to identify item relations and similarities. On the one hand, this opens up the possibility of selecting the appropriate variables and items for research questions within and across cohorts or for clinical questions in the medical setting. On the other hand, it has the potential to uncover new, previously undiscovered connections, constructs, and concepts. The suggested process allows not only pinpointing overlapping concepts but also grasping the nuanced relationships between individual survey items. By leveraging data manipulation and visualization libraries, it can harness the power of a search function. For this reason, our framework integrates NLP techniques and cosine similarity measurements [17]. These advanced tools facilitate the exploration of fine-grained relationships between individual items within different sources with language-based information from research and clinical instruments.

**Figure 9.**
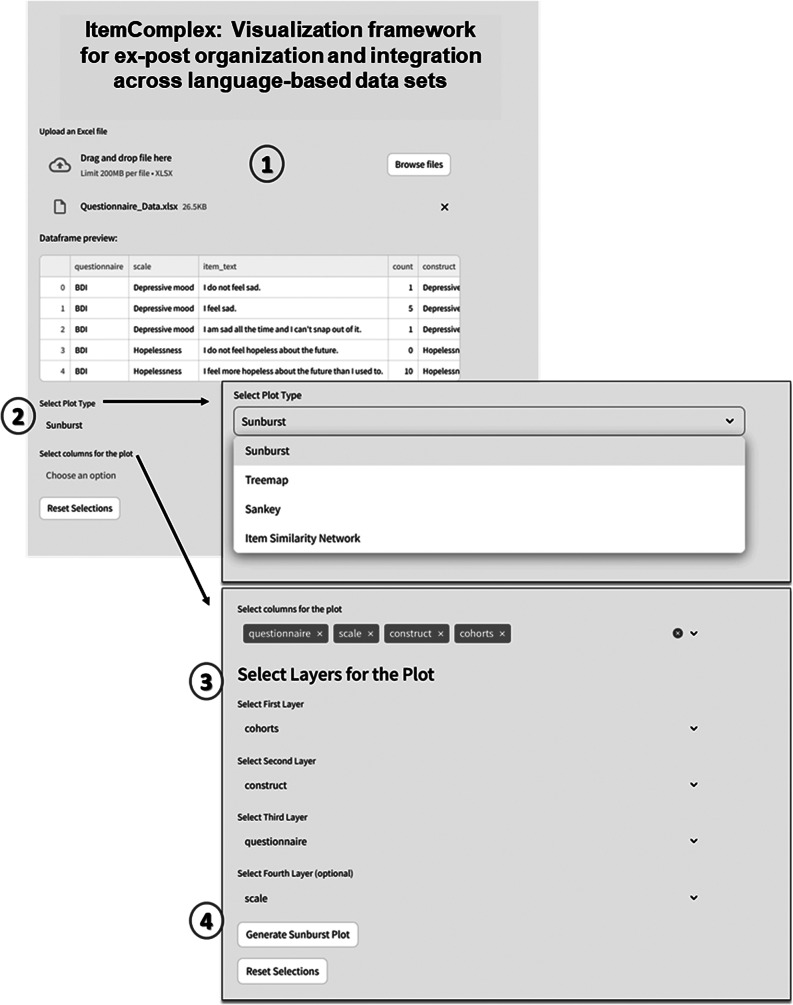
Interface of ItemComplex framework. *Note:* 1 – Data upload and preview: In this step, users can upload an Excel file containing multi-item instrument data (e.g., questionnaires such as BDI, CES-D, etc.). The data preview table displays the uploaded dataset, showing columns like “questionnaire,” “scale,” “item text,” and “construct.” 2 – plot type selection: Users can choose from multiple plot types to visualize the data, such as “Sunburst,” “Treemap,” “Sankey,” or “Item Similarity Network.” 3 – column selection for plot layers: Users can select different columns from the dataset to define the hierarchical layers for the visualization. For example, “cohorts” could be selected as the first layer, followed by “construct,” “questionnaire,” and optionally “scale.” 4 – generate plot: After selecting the plot type and columns, the “Generate Plot” button is used to create the interactive visualization. The system then processes the data and displays the sunburst plot, which visually represents the hierarchical structure of the selected attributes.

*ItemComplex feasibility and usability for data from different cohort studies.* The ItemComplex framework enables comprehensive data integration by allowing users to import and combine data from multiple large-scale (cohort) studies. In practice, ItemComplex is available in two forms: (1) an interactive interface that works “out of the box” and requires no programming experience and (2) a suite of Python scripts for users who wish to integrate or adapt the code for specialized scenarios. The interactive option provides intuitive data upload, visual exploration via sunburst, TreeMap, or Sankey diagrams, and basic parameter tuning; meanwhile, the underlying scripts allow deeper customization (e.g., selecting specific similarity metrics or modifying plotting routines). With either mode of use, the framework offers multiple analysis options suitable for diverse research questions, including advanced similarity checks across items and constructs. The *ItemComplex* app enables comprehensive data integration by allowing users to import and combine data from different large-scale (cohort) studies (see Figure 9). It offers multiple analysis options with these multiplex datasets, while the focus on a specific research question can be considered. Moreover, the app facilitates advanced similarity checks across items and constructs computing and visualizing similarities between items across different datasets, helping to identify common and distinct patterns and clusters. The interactive features, including visualizations through sunburst, TreeMap, and Sankey diagrams, provide intuitive ways to explore and understand the relationships and overlaps in the data and to avoid errors by manual tracking. This also includes the integration of customizable insights through a specific data layer structure as well as the export functions supporting further advanced analysis with external sources and the sharing of findings.

*Identification of overlapping variables within and across studies and cohorts through data manipulations and visualization tools.* To address the challenge of identifying overlapping psychometric constructs and/or subscales within and across studies, we adapted a Python-based script that utilizes powerful libraries for data manipulation and visualization. By employing the Pandas [18], HoloViews [19], and Plotly libraries, we created a code snippet structured along different content topics (e.g., clinical symptoms of anxiety, depression, or personality) to facilitate the analysis of the diverse psychometric instruments. We also implemented an analogous visualization process of these structural analyses to offer valuable insights into the selected content topics per study and across studies. The full code description and implementation details of the *ItemComplex* app framework, including data preprocessing and visualization techniques, are thoroughly described in the supplementary materials.

**Figure 10.**
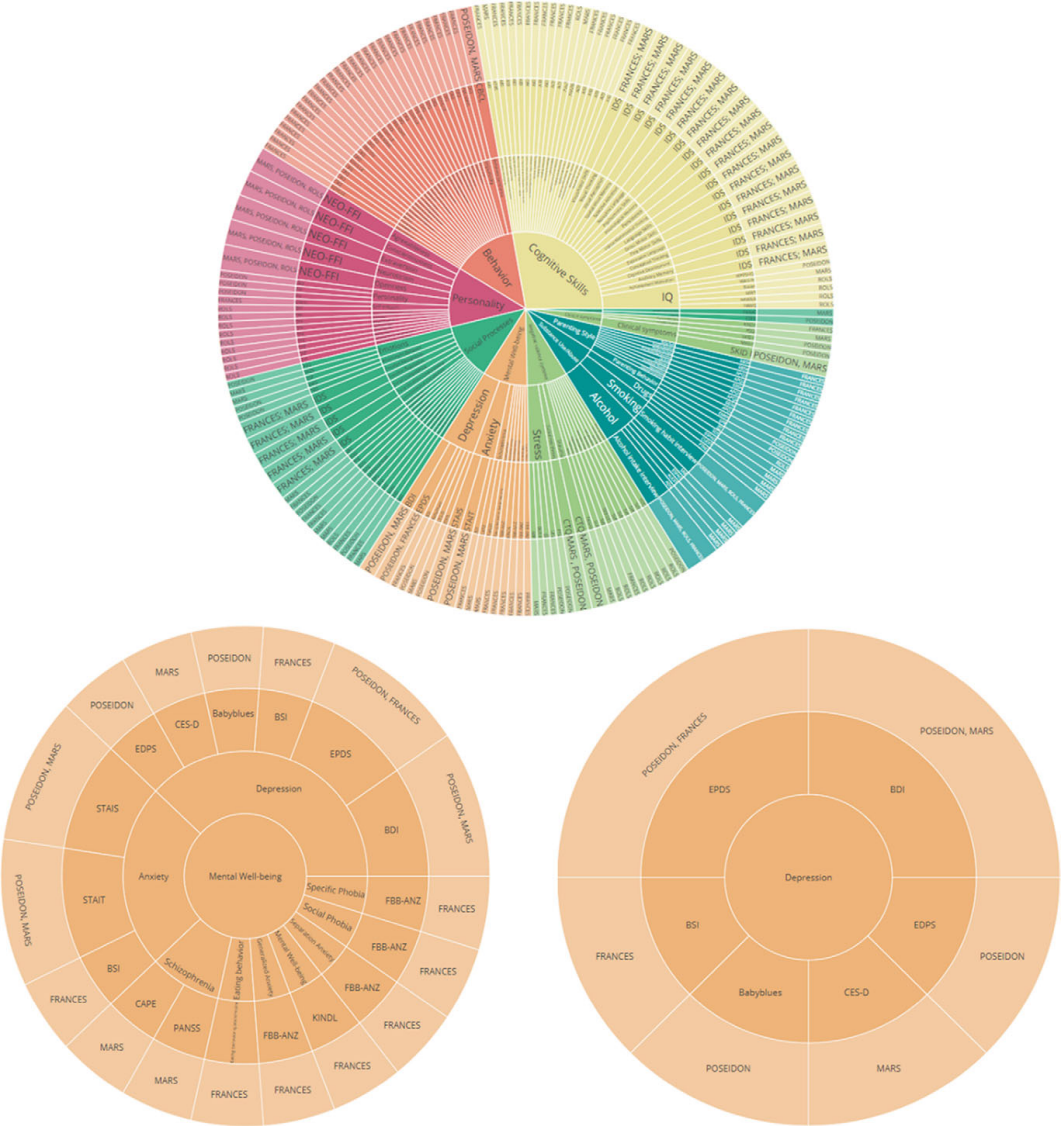
Sunburst plots of overlapping constructs across different studies. The plot represents different layers that provide information about overlapping questionnaires, constructs/subscales across different studies. Similarly, the adaptable nature of the visualization allows for a personalized sequencing of the layers (for example starting with cohort-specific information within the central circle).

**Figure 11.**
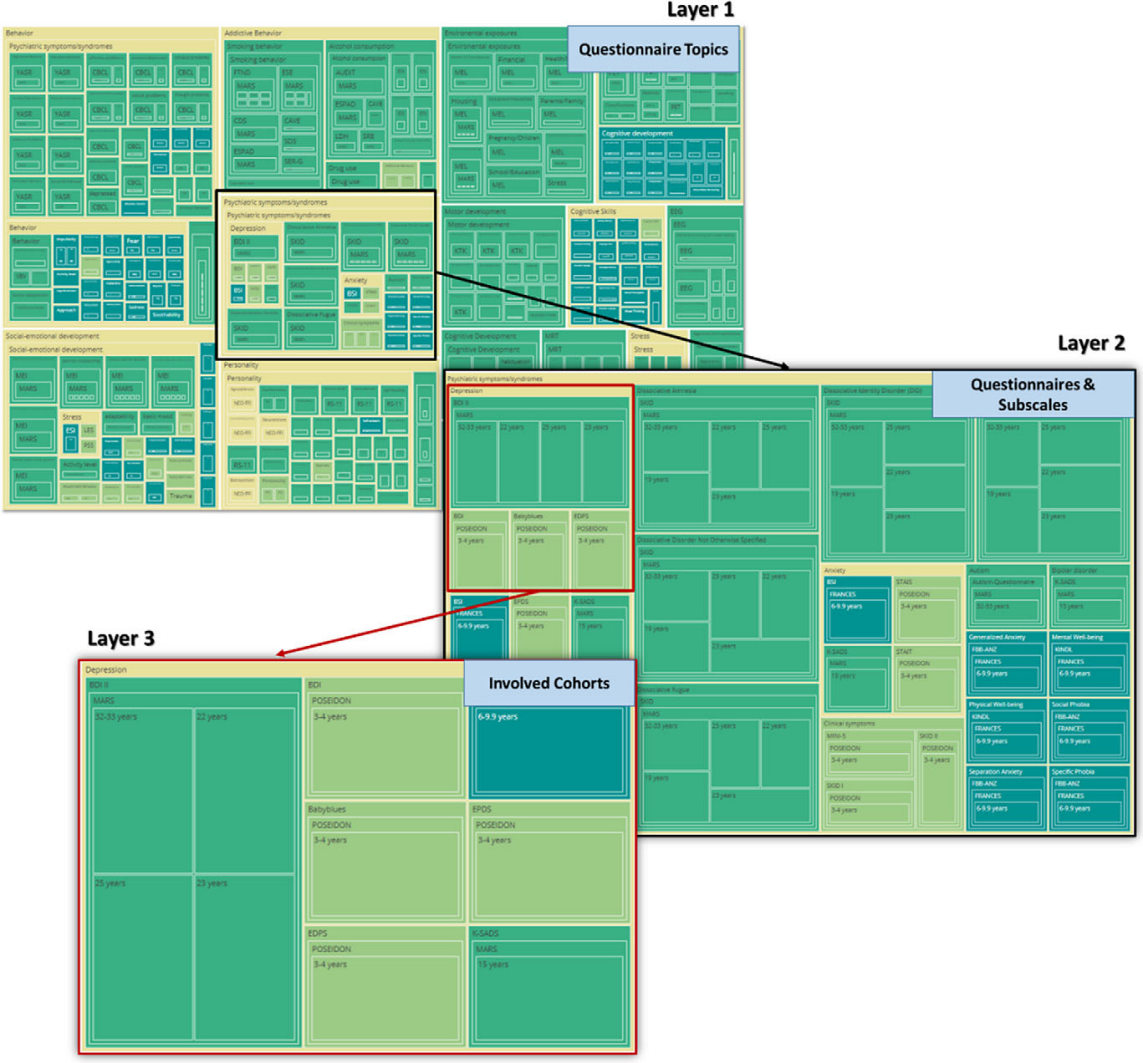
TreeMap plots of overlapping constructs across different studies. The plot showcases hierarchical layers that illustrate the overlap of questionnaires and constructs/subscales across various studies. The adaptable design of the visualization allows for customizable sequencing of the layers, such as beginning with cohort-specific information at the central node, enabling tailored insights into the data’s structure and relationships.

Specifically, the sunburst plot is composed of nested circles, each representing a hierarchical level of data. Starting from the center, the innermost circle signifies the highest level of categorization, and subsequent circles moving outward depict more specific categories (Figure 10). With the code snippet, we utilized the ’path’ parameter to define the hierarchy and categories such as ’Level’, ’Construct’, ’Questionnaire’, and ’Cohort’. We also employed a color scheme, with colors indicating various levels or attributes, aiding in quick comprehension and differentiation of constructs/subscales. The interactive nature of the plot enhances its utility. Users can interact with the visualization by hovering over segments to reveal tooltips with detailed information. Clicking on segments allows for zooming in and out, navigating deeper into the hierarchy, or returning to broader views. This interactive capability empowers the exploration of big datasets. Another visualization feature, the TreeMap plot (Figure 11), offers a comprehensive overview of the data with opportunities to add other information regarding cohorts, such as sample sizes or age groups. The TreeMap plot utilizes nested rectangles to represent hierarchical data, with the size of each rectangle proportional to a specified metric, such as count or value. This allows for an efficient use of space and a clear visual representation of the relative importance or prevalence of each construct category. Like the sunburst plot, the TreeMap is interactive, with tooltips providing detailed information on hover and the ability to click on rectangles to explore deeper levels of the hierarchy. The TreeMap’s customization options make it possible to visualize additional layers of information, such as time points of assessment or different versions of questionnaires, enhancing the depth and utility of data analyses.

*Exploring item-level relations within and across studies and cohort data through semantic similarity analyses and representing in network and graph-based visualizations.* To delve into item-level relationships and achieve a more granular perspective of the psychometric data and breaking up standard associations, we developed several algorithms. These algorithms preprocess textual data from survey items, convert it into numerical representations using TF-IDF, and then calculate cosine similarity to identify items with similar meanings. The results are visualized in network-based graphs, where nodes represent items and edges connect those with high similarity, revealing clusters of related items and their constructs. This allows an overview of single-item representations within and across cohorts, and detection of new content-based constructs and concepts. Using a data mapping technique, we implemented an algorithm to enable item selections for data normalization and standardization to bring the data to a common level and thus inform important steps in the data harmonization process. This is particularly useful for very diverse cohort data that do not allow for simply combining (sub)scales of instruments. For example, if we consider the symptoms of depressed mood, which are measured across different psychometric instruments like the Brief Symptom Inventory (BSI), the Beck Depression Inventory (BDI), and the Center for Epidemiologic Studies Depression Scale (CES-D), we see that each of these instruments includes items that capture facets of depression, but they differ in the number of items, phrasing, and focus areas. The BSI is a shorter instrument that contains items with a focus on general psychological distress such as “Feeling no interest in things.”, but also offers a more comprehensive view of depression with specific items addressing cognitive and emotional symptoms like “I feel sad” and “I blame myself for everything.” Meanwhile, the CES-D mainly covers both emotional and physical aspects of depression, with items like “I felt that everything I did was an effort” and “I could not get going.” By applying our algorithm, we break down the textual contents of different items into numerical representations using TF-IDF. This allows us to calculate the cosine similarity between items across the instruments, identifying which items are semantically similar even if they use different wording. Two items from two different questionnaires that show high similarity indicate that these two items assess the same underlying concept, although having variations in language. Through network-based visualizations, we can see clusters of related items, where nodes represent the items and edges connect those with a cosine similarity above a certain threshold (e.g., 0.5). This reveals how different instruments overlap in their assessment of depression. For instance, we can identify that items related to depressed mood cluster together across the BSI, BDI, and CES-D, but the CES-D introduces additional clusters related to somatic complaints not covered as extensively in the BDI or BSI. This establishes a reliable foundation for deciding whether cohort data from different instruments can be combined for analysis without introducing significant bias in the interpretation of the results. Alternatively, it may indicate that only certain subscales or individual items should be merged to address a specific research question accurately. The framework includes an extension of the above-described structure to individual survey items, stripping them of punctuation and stop words, and transforming them into tokens. The proposed methodology begins by preprocessing the textual content of items from different questionnaires. This preprocessing involves converting the text to lowercase, removing punctuation, tokenizing the text into words, and eliminating common stop words (words like “the,” “and,” “of,” “in,” “is,” etc.). Next, we grouped the items based on their corresponding questionnaires. This grouping allowed us to focus on identifying semantic similarities within the same context. By applying a TF-IDF vectorization technique [20, 21], we transformed the textual data into numerical representations that highlight the importance of words within each item and questionnaire. TF-IDF scores capture the essence of the vocabulary while downplaying common words. We then applied cosine similarity, a measure of how alike two vectors are in direction and magnitude to compute the similarity between TF-IDF vectors representing different items. For our use case data, we calculated cosine similarity across the items within each questionnaire. A cosine similarity matrix was generated, where higher values indicate stronger semantic similarities. This ensured the discovery of items that share semantic content but may use different phrasing. We implemented innovative visualization tools, such as interactive charts to navigate through the intricate web of relationships at the molecular level. For all these steps, we applied the NLTK and NetworkX Python libraries.

**Figure 12.**
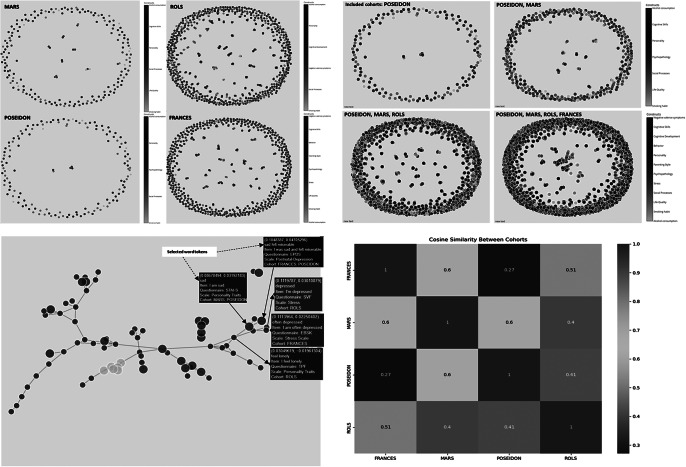
Network visualization for item similarity identification. Spring layout algorithm, positioning items with higher similarity in closer proximity (cosine similarity cut-off value =.50), colors correspond to the associated construct, facilitating the identification of clusters wherein related items sharing the same construct are visually discernible. The hover tooltip provides contextual information related to the specific element being hovered over. The tooltip displays information such as selected tokens, the actual text of the survey item, the originating questionnaire, the associated scale or subscale, and the corresponding cohort, and the values in the first line present the weight value associated with the edge, which quantifies the similarity between connected survey items.

Our proposed network is composed of nodes, each representing a distinct survey item, and edges denoting connections between items that exhibit a cosine similarity above a threshold of 0.5 (Figure 12). The spring layout algorithm is employed to arrange nodes, placing items with higher similarity closer together. Nodes are visualized as circles, with sizes corresponding to the frequency count of the respective survey items. The colors of the nodes are determined by the construct to which each item belongs. This coloring scheme provides a visual means to identify clusters of related items that share the same construct.

Hovering over a node reveals essential details such as the item’s label, original identifier, associated questionnaire, scale value, and cohort information, offering researchers an in-depth contextual understanding of each item (Figure 12). Edges between nodes represent significant similarities between items, with thicker edges indicating higher cosine similarity values. The thickness of the edges offers a clear depiction of the strength of relationships between items, aiding in the identification of strongly related pairs.

The generated exploratory analyses pipelines and related plots enable the identification of clusters of related items and the discovery of hidden similarities, through a smooth, quick, and easy-to-understand construct visualization. The proposed network analyses thus offer a novel perspective on item relationships by providing an intuitive and comprehensive representation of diverse and big psychometric datasets. It facilitates the identification of emerging patterns and informs further analysis.

**Figure 13.**
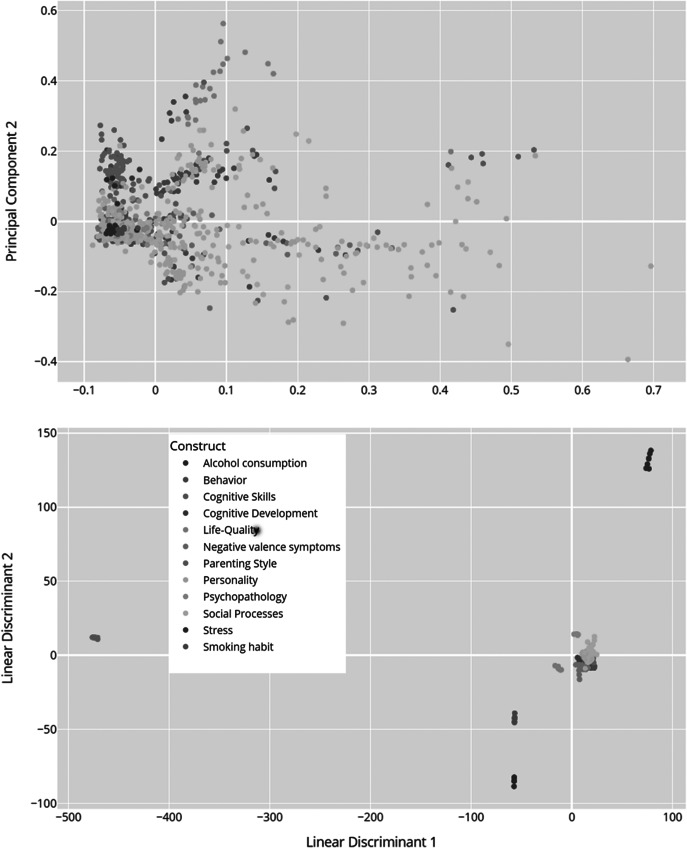
Dimensionality reduction of TF-IDF features. The PCA scatter plot (above) displays items projected onto the first two principal components derived from the TF-IDF feature matrix of preprocessed text data. These components capture the greatest variance in the high-dimensional space. Each data point represents an individual item and is color coded according to its associated construct (e.g., theoretical category). The dispersion of points reflects overall variability in semantic content, while clustering suggests underlying similarities among items with the same construct label. The LDA scatter plot (below) illustrates a supervised projection of the same TF-IDF features, with the axes representing the first two linear discriminants. This analysis seeks to maximize the separation between the predefined construct classes. Each point in the plot corresponds to an item, and the colors denote its construct label. Compared to PCA, LDA emphasizes class differences by concentrating on between-class variance, thereby highlighting the distinct clusters corresponding to the different constructs.

In addition to generating the node-link diagram using a spring layout and a manually set similarity threshold, we also experimented with dimension reduction methods (e.g., Principal Component Analysis, PCA) to place nodes in a lower-dimensional space. This approach avoids relying on a discrete similarity threshold to draw edges; instead, items are displayed in a continuous scatter plot that reflects their relative distances. We present the PCA-based scatter plot, along with linear discriminant analysis (LDA) visualizations (Figure 13) for additional insights into underlying item-level patterns. While dimension reduction can offer a clearer global overview of relationships among items, particularly when the precise choice of threshold is a concern, we found the threshold-based node-link diagram to be valuable for highlighting strong conceptual overlaps or clusters of items that exceed a certain similarity. By adjusting the threshold, researchers can tailor the network view to focus on more conservative or more inclusive relationships as needed.

*Run time optimization for large datasets.* For handling large (cohort) datasets efficiently, our app system incorporates advanced runtime optimization techniques. To manage the computational load, we employed scaling to control for large data sizes while minimizing processing time. This includes the optimization of data preprocessing steps with text tokenization and TF-IDF vectorization and the leveraging of sparse matrix representations to reduce memory usage. Additionally, we implemented parallel processing features distributing tasks across multiple processors to speed up similarity calculations and matrix operations. By using these strategies, our system maintains high performance and responsiveness, even when dealing with extensive and complex datasets. This ensures quick analyses and visualizations without encountering significant delays or performance issues.

## Discussion

With the *ItemComplex* app, we present a comprehensive Python-based interactive tool capable of addressing the challenges associated with organizing, structuring, and analyzing large and diverse psychometric datasets at the multi-instrument level that is flexibly applicable in a variety of settings. The framework includes a suite of algorithms for data structuring according to content topics, complemented by powerful visual tools such as sunburst and TreeMap plots. The tool enables manipulating and visualizing big and complex cohort data to illustrate and help understand the order, overlap, and assignment of constructs/subscales within and across different studies. By presenting data hierarchically, users can navigate through various levels of granularity, gaining insights into the distribution and interconnections of constructs/subscales.

With the principle of *ItemComplex*, there is a potential to be suitable for multiple data modalities and diverse scientific questions. To explore item-level relations within and across studies, the algorithms included NLP techniques and cosine similarity measurements. With the network analyses and the related visualization process, users can identify semantic similarities between individual survey items to have a more detailed perspective on psychometric data including comprehensive insights into potentially hidden patterns and connections. This capability aids in building new constructs and deepening the understanding of psychological and medical concepts.

*ItemComplex* adds to and is different from currently available harmonization tools [22, 23] in that it focuses on visualization techniques integrating advanced semantic analysis and network-based approaches specifically designed for large and divergent psychometric data. Moreover, it links this visualization with features to use the visualized information to directly perform preliminary analyses. Through this, it adds another layer emphasizing a deeper, content-based approach and allows a fast and valid first overview of complex language-based datasets from diverse resources. This approach not only facilitates the alignment of diverse datasets but can also help uncover hidden patterns and connections, offering a richer, more detailed understanding of the data that goes beyond basic numerical alignment.

By integrating data from diverse studies, *ItemComplex* enables cross-study analyses, construct validation, and a holistic comprehension of complex information. This capability is particularly valuable for large-scale consortia, but can also be integrated into clinical contexts where information from documentation sheets needs to be evaluated and used for further processes. The relevance of *ItemComplex* can be further underscored by comparing it with approaches and challenges identified in recent literature. For instance, the problem of data imbalance discussed by Khan et al. [24] highlights the critical need for balanced and accurate datasets for reliable research outcomes. *ItemComplex*, through its hierarchical data structuring and visualization capabilities, can help mitigate difficulties related to data imbalance by providing a more nuanced understanding of data distribution and relationships. By employing advanced techniques like NLP and cosine similarity, it ensures a detailed and balanced representation of data, enhancing accuracy and reliability.

Finally, regarding practical usage, ItemComplex can be run either through a point-and-click interface or by editing the underlying Python scripts. In the interactive version, tasks such as uploading data, selecting among various visualizations (sunburst, TreeMap, or network graphs), and filtering items can be done entirely via user-friendly menus. This makes it straightforward for researchers and clinicians with limited coding experience to explore large, complex datasets. Meanwhile, for those with more specialized needs, the Python code (accompanied by step-by-step documentation) allows deeper customization, such as incorporating additional similarity metrics or tailoring visual layouts to specific study requirements. To facilitate diverse user needs and analytical goals, ItemComplex offers several visualization types, including sunburst and TreeMap plots. Although these two layouts both depict hierarchical data, each has distinct strengths and appeals to different user preferences: The sunburst layout organizes hierarchical structures into concentric rings, making “parent-child” relationships visually explicit. Users can drill down through each layer simply by clicking the rings, thereby navigating deeper into nested categories. Sunbursts tend to be more intuitive for revealing the hierarchical flow and connections between dataset segments. By contrast, TreeMaps rely on nested rectangular areas to represent hierarchical relationships, allowing users to visually explore proportions within the data. The size of each rectangle corresponds to its relative share within the overall dataset. This design readily highlights quantitative differences, which can be less apparent in sunburst diagrams. In this way, ItemComplex remains accessible as an “out-of-the-box” solution while still offering advanced possibilities for users who wish to adapt the tool to their unique data scenarios.

Some limitations should be considered. Although the consortium-based integration has demonstrated that ItemComplex can be helpful in real-world data harmonization tasks, we did not conduct a dedicated user study with structured protocols (e.g., measuring completion times or error rates). This reliance on practical use scenarios provided valuable but less systematically quantifiable feedback. As a result, future work might complement the consortium-based feedback with more formal usability testing and direct comparisons to alternative visualization tools.

## Code availability

Interactive visualizations used in this paper are available under https://karinajanson.github.io/imacmind/sunburst/ for sunburst plot, https://karinajanson.github.io/imacmind/sunburst/ for TreeMap visualization, and https://karinajanson.github.io/imacmind/itemnetwork/ for item similarity network. If researchers are interested in using the ItemComplex app, which runs with a user interface following the Streamlit application, for their own dataset, the corresponding author can be contacted. The rationale is that we will implement a secure data structure, even though no personally identifiable information will be uploaded to the platform. Additionally, this will enable us to evaluate user experiences, allowing for continuous updates based on user needs and advancements in the field.
